# Expanding the spectrum of neonatal‐onset AIFM1‐associated disorders

**DOI:** 10.1002/acn3.51876

**Published:** 2023-08-29

**Authors:** Alberto A. Zambon, Daniele Ghezzi, Cristina Baldoli, Gianni Cutillo, Katia Fontana, Valentina Sofia, Maria Grazia Patricelli, Alessia Nasca, Stefano Vinci, Ivana Spiga, Eleonora Lamantea, Giovanna F. Fanelli, Maria Grazia Natali Sora, Rosanna Rovelli, Antonella Poloniato, Paola Carrera, Massimo Filippi, Graziano Barera

**Affiliations:** ^1^ Unit of Neurology San Raffaele Scientific Institute Milan Italy; ^2^ Neuromuscular Repair Unit, Institute of Experimental Neurology (InSpe), Division of Neuroscience IRCCS Ospedale San Raffaele Milan Italy; ^3^ Medical Genetics and Neurogenetics Unit Fondazione IRCCS Istituto Neurologico Carlo Besta Milan Italy; ^4^ Department of Pathophysiology and Transplantation University of Milan Milan Italy; ^5^ Department of Neuroradiology San Raffaele Scientific Institute Milan Italy; ^6^ Neurophysiology Service San Raffaele Scientific Institute Milan Italy; ^7^ Department of Neonatology San Raffaele Scientific Institute Milan Italy; ^8^ Laboratory of Genomics and Clinical Genetics San Raffaele Scientific Institute Milan Italy; ^9^ Unit of Genomics for Human Disease Diagnosis San Raffaele Scientific Institute Milan Italy; ^10^ Vita‐Salute San Raffaele University Milan Italy

## Abstract

**Objectives:**

Pathogenic variants in *AIFM1* have been associated with a wide spectrum of disorders, spanning from CMT4X to mitochondrial encephalopathy. Here we present a novel phenotype and review the existing literature on *AIFM1*‐related disorders.

**Methods:**

We performed EEG recordings, brain MRI and MR Spectroscopy, metabolic screening, echocardiogram, clinical exome sequencing (CES) and family study. Effects of the variant were established on cultured fibroblasts from skin punch biopsy.

**Results:**

The patient presented with drug‐resistant, electro‐clinical, multifocal seizures 6 h after birth. Brain MRI revealed prominent brain swelling of both hemispheres and widespread signal alteration in large part of the cortex and of the thalami, with sparing of the basal nuclei. CES analysis revealed the likely pathogenic variant c.5T>C; p.(Phe2Ser) in the *AIFM1* gene. The affected amino acid residue is located in the mitochondrial targeting sequence. Functional studies on cultured fibroblast showed a clear reduction in AIFM1 protein amount and defective activities of respiratory chain complexes I, III and IV. No evidence of protein mislocalization or accumulation of precursor protein was observed. Riboflavin, Coenzyme Q10 and thiamine supplementation was therefore given. At 6 months of age, the patient exhibited microcephaly but did not experience any further deterioration. He is still fed orally and there is no evidence of muscle weakness or atrophy.

**Interpretation:**

This is the first *AIFM1* case associated with neonatal seizures and diffuse white matter involvement with relative sparing of basal ganglia, in the absence of clinical signs suggestive of myopathy or motor neuron disease.

## Introduction

The *AIFM1* gene encodes for the apoptosis‐inducing factor mitochondrion‐associated 1 and is located on the X chromosome at Xq26.1. AIFM1 is a 67‐kDa homodimeric flavoprotein that usually resides in the inner mitochondrial matrix. When cleaved to its soluble form upon apoptotic stimuli, AIFM1 translocates to the nucleus where it contributes to caspase‐independent cell death by inducing DNA fragmentation, chromatin condensation and production of apoptogenic proteins.^1^ Moreover, AIFM1 plays an important role in oxidative phosphorylation (OXPHOS) by providing reductase activity^2^ and participating in the biogenesis of respiratory chain (RC) complexes.^3^, ^4^

The spectrum of clinical manifestations that arise from *AIFM1* mutants is wide and may be the result of a defect in more than one of its several functions.^5^

Childhood‐onset conditions include Cowchock syndrome (i.e. axonal neuropathy associated with deafness and cognitive impairment – CMTX4, MIM#310490),^6^, ^7^, ^8^ cerebellar ataxia with peripheral neuropathy and deafness,^9^, ^10^ isolated deafness (MIM#300614),^11^ and hypomyelinating leukodystrophy with spondylometaphyseal dysplasia (H‐SMD, MIM#300232).^12^, ^13^

Moreover, patients with mutations in *AIFM1* may have a congenital or infantile encephalomyopathy disorder associated with combined oxidative phosphorylation deficiency (i.e., COXPD6, MIM#300816). The phenotype is usually characterized by a severe mitochondrial encephalopathy variably associated with systemic features (e.g., cardiomyopathy^14^), lactic acidosis, cerebral ventriculomegaly,^15^ seizures, and lower motor neuron degeneration.^5^, ^16^, ^17^

In this report, we present a novel association between a likely pathogenic variant in *AIFM1* affecting the mitochondrial targeting sequence (MTS) and a clinical presentation characterized by neonatal seizures and diffused white matter changes on brain magnetic resonance imaging (MRI). To our knowledge, this is the first report describing such a combination of findings, thus expanding the spectrum of *AIFM1*‐related phenotypes. We also review in detail the existing literature on *AIFM1*‐related disorders and associated clinical phenotypes.

## Methods

### Clinical investigations

Investigations including video‐electroencephalography (EEG), brain MRI, brain MR spectroscopy (MRS), echocardiogram and abdomen ultrasound were performed according to standard techniques.

### Genetic analysis

Genomic DNA was isolated from whole blood with a Maxwell‐Promega protocol. Clinical exome sequencing (i.e., TruSight One Expanded 6794 genes panel and DNA Prep with Enrichment by Illumina) was performed using the NextSeq500 Illumina platform and primary analysis with the Dragen software.^18^ After filtering of common variants with general population frequency >1%, an unbiased prioritization of variants was performed using HPO phenotypic descriptors; in addition, a selection of 60 genes involved in epileptic encephalopathies was analyzed, using the bioinformatic tools enGenome‐eVai and Alamut‐Qiagen as well as the databases: NCBI dbSNP, gnomAD, ClinVar, LOVD, PubMed, Mastermind‐Qiagen. Variants were classified according to ACMG‐AMP criteria.^19^ Reported variants were confirmed by Sanger sequencing. A CGH‐Array was performed using oligo genome‐wide GenetiSure Cyto 4x180K CGH platform.

### Cell culture, histochemical analyses and protein assessment

All human tissues in this study were acquired and processed under appropriate consent and institutional research ethics cover. Primary cultures of fibroblasts were established from a punch skin biopsy of the thigh.

Mitochondrial RC complex activities were measured using standard spectrophotometric methods in digitonin‐treated skin fibroblasts,^20^ and normalized to citrate synthase activity, an index of mitochondrial content in the analyzed specimens.

Steady‐state protein levels of AIFM1 protein were assessed by SDS‐polyacrylamide gel electrophoresis (SDS‐PAGE) in cell lysates from the patient and controls as previously described.^16^ Immunoblotting was carried out using a rabbit polyclonal anti‐AIF antibody (Millipore) and a mouse monoclonal anti‐GAPDH (Millipore) followed by species appropriate HRP‐conjugated secondary antibodies. Fibroblasts grown in standard glucose medium were stained with the specific antibody against AIFM1 and with a mitochondrial dye (Mitotracker) and examined by fluorescence microscopy on a confocal microscope (Leica TSC‐SP8).^21^

### Standard protocol approvals, registrations, and patient consents

Patients provided written informed consent for genetic analysis and for the use of their coded data for research purposes, as approved by the Ethics Committee of the IRCCS Ospedale San Raffaele.

### Data availability

Clinical and genetic anonymized data are available from the corresponding author and from the Laboratory of Genomics respectively, upon reasonable request.

## Results

### Patient description

The patient is a 6‐month‐old boy, first child of non‐consanguineous parents with no family history of neurological diseases or spontaneous abortions. He was born at term by emergency C‐section due to not reassuring cardiotocography. Pregnancy was otherwise uneventful. APGAR score was 6 at the 1st and 5th minute, his weight 2670 g. Amniotic fluid was stained with meconium. Upper respiratory airways aspiration and T‐piece ventilation were performed and nasotracheal tube positioning with mechanical ventilation was implemented after 5 min. Venous gas analysis initially revealed a mild funicular acidosis (pH 7.17, BEB −6.1 mmol/L, pCO2 64 mmHg, lactate levels 12 mmol/L). The patient was not deemed eligible for therapeutic hypothermia by the neonatologist on call (amplitude‐integrated electroencephalography was not performed).^22^, ^23^ At 3 h of life, accidental extubation occurred and he was then supported with high‐flow nasal cannula. There were no signs of infection, including inflammatory index (CRP) curve, blood cultures, chest X‐ray and pharyngeal swab.

**Figure 1:**
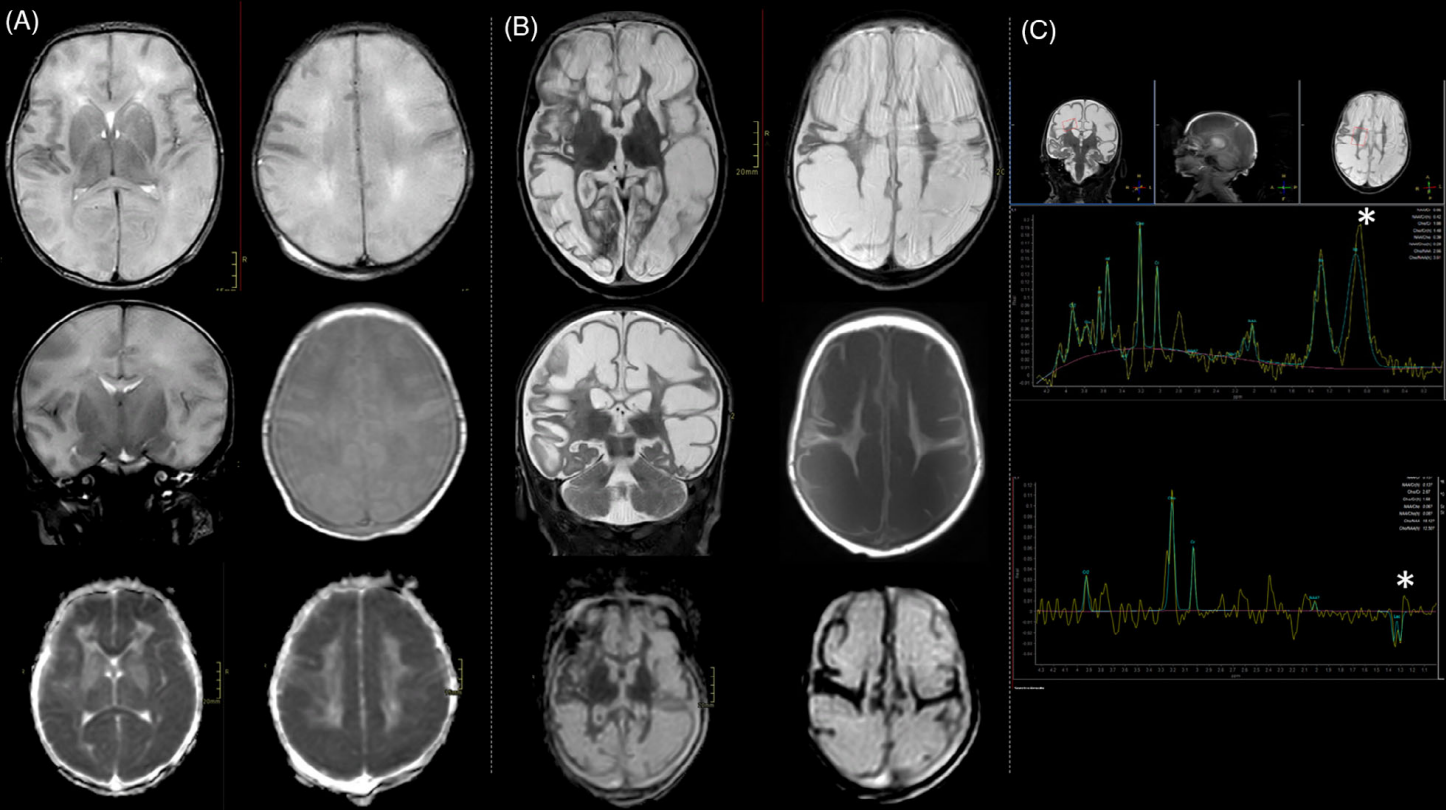
(A) Brain MRI on day 1 (Transverse and coronal T2 WI, transverse T1 WI, DWI ADC) showed brain swelling with T2 diffuse white matter mild hyperintensity, swollen appearance of the cortical gyri with flattening of the cortical sulci and apparent thinning of the cortex. DWI ADC (bottom) showed significantly restricted diffusivity of the white matter (mean 0,4 mm^2^ sec). (B) Brain MRI on day 30 (Transverse and coronal T2 WI, transverse T1 WI, DWI ADC) showed evolution toward multicystic encephalomalacia with marked T2 hyperintensity and T1 hypointensity of the white matter. DWI ADC diffusivity considerably increased (3 mm^2^ sec). (C) MR spectroscopy (short TE‐31 ms– at the top and long TE‐144 ms– at the bottom) exhibited high lactate peak, with typical doublet inverted at long TE (asterisks). There was also an increased peak of choline and reduction of NAA compared to reference values for age.

**Figure 2:**
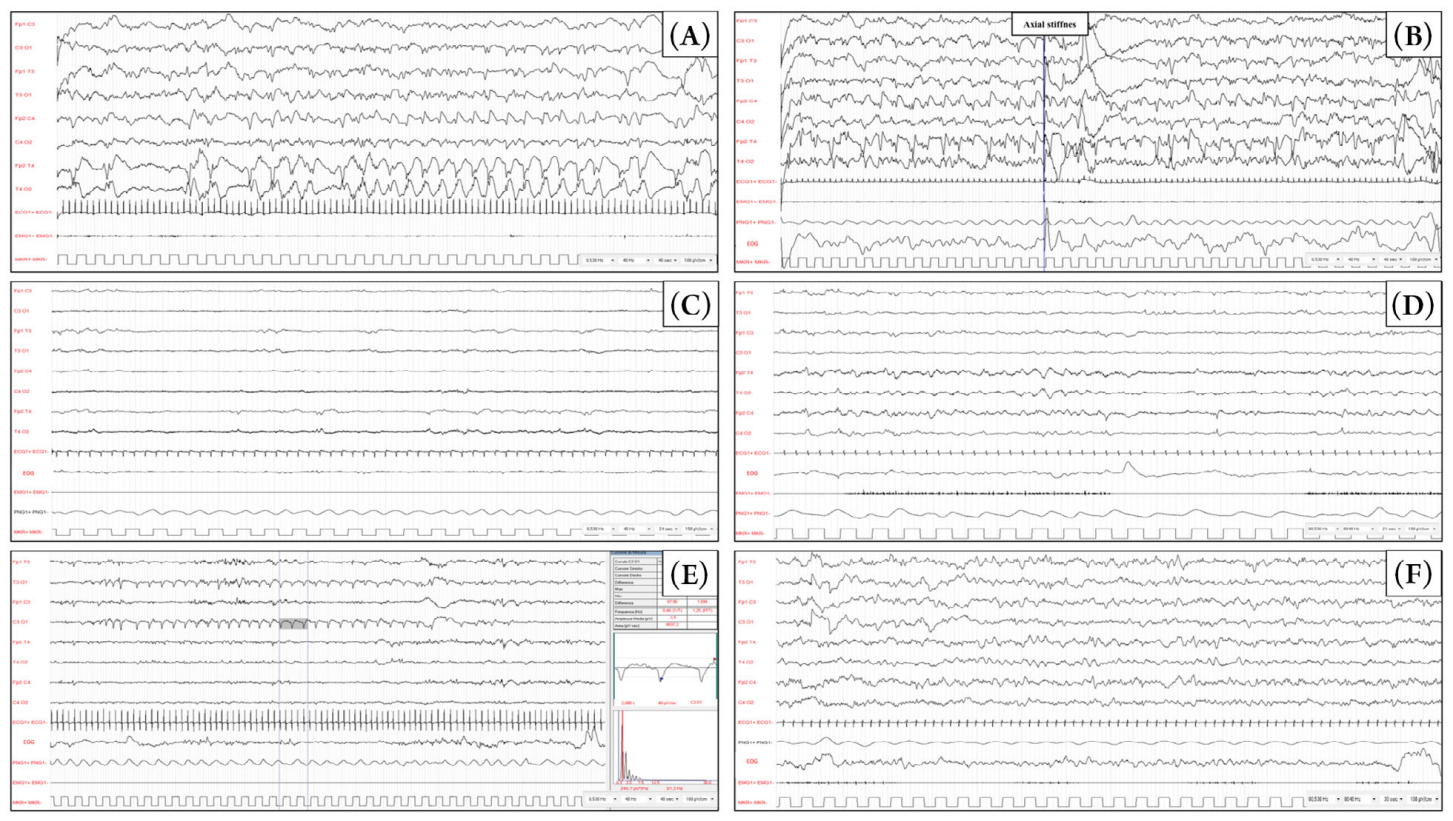
EEG tracings were acquired using a restricted neonatal montage including eight scalp electrodes (Fp2, C4, T4, O2, Fp1, C3, T3, O1), the ECG, electrooculography (EOG), pneumogram (PMG). A surface electrode was placed over the mylohyoid muscle (EMG). Acquisition parameters are shown in the bottom right corner of each panel. (A, B) EEG 7 h after birth during quiet sleep showed seizure originating from both hemisphere activities with continuous rhythmic epileptic discharges. Panel A shows ictal activity over the right temporal regions without overt clinical correlation. Panel B shows seizure activity, originated from the left hemisphere, diffusing over the right hemisphere while the patient displayed axial stiffness. (C) EEG 1 week after birth during quiet sleep, upon suspension of midazolam, showed a diffused slowing of the background and global suppression of cerebral activity, with synchronous and asynchronous epileptic discharges over the temporal regions of both hemispheres. (D) The interictal EEG 30 days after birth showed an alteration of the general organization in sleep, with no recognizable physiological sleep figures. Synchronous and asynchronous epileptic discharges can be observed over the temporal regions with distinct right predominance. (E) Ictal EEG 50 days after birth; during quiet sleep, a 30‐s sequence of periodic, 1–1.5 Hz, sharp waves could be observed over the left temporal region, without clinical correlate. (F) follow‐up EEG at 6 months. No sleep figure is recognizable and slow waves and occasional sharp waves are present over the temporal regions of both hemispheres, with a right predominance.

After 6 h, generalized hypertonia, hyperexcitability and cycling movements of the limbs occurred. Video‐EEG demonstrated a disorganized background with multifocal electroencephalographic and electroclinical seizures both during wakefulness and sleep, later evolving into a burst‐suppression pattern (Fig. 2A, B). Seizures were resistant to phenobarbital, phenytoin and levetiracetam so midazolam infusion was started. A partial reduction in seizure frequency was observed after the administration of pyridoxine, although there might be an underlying role of concomitant polytherapy. Brain MRI (day 1) revealed prominent and diffuse brain swelling of both hemispheres with widespread signal alteration (highly T2 hyperintense/T1 hypointense and with diffusion restriction) suggesting cytotoxic oedema in large part of the cortex and of the thalami, with sparing of the basal nuclei (Fig. 1A).

Blood test initially revealed increased CPK levels (1671 IU/L, normal values: 20–195), abnormal liver and renal function tests (AST/ALT >5 × ULN), and high lactate, but later normalized. Uric acid and ammonia levels were normal. Extended metabolic screening including very long‐chain fatty acids was negative. Repeated echocardiograms and abdomen ultrasound were unremarkable.

On first neurological examination performed after the initiation of antiepileptic drugs (day 1), the clinical picture was mainly characterized by mild lethargy, poor spontaneous movements, hyperexcitability, and mild pyramidal signs. We did not observe facial dysmorphic features; head circumference and cranial nerves were normal. Trunk and limb posture was normal and there was no muscle weakness.

EEG monitoring showed a progression to suppression‐burst pattern in the following days (Fig. 2C). Subsequent MR follow‐up examinations, including the most recent one performed after a 30‐day interval, revealed regression of diffusivity restriction accompanied by an increase in diffusion values (Fig. 1B, bottom). Furthermore, there was a significant cystic degeneration accompanied by a reduction in the swelling of the cerebral gyri, although white matter signal alterations persisted, exhibiting higher signal intensity on T2‐weighted images and lower signal intensity on T1‐weighted images (Fig. 1B, top and medium panel). Diffuse cortical thinning was observed. Notably, along with such initial encephalomalacic changes, the spectroscopy study continued to exhibit pathological findings, characterized by a prominently elevated lactate peak (Fig. 1C).

Based on the results of the analysis of the mitochondrial respiratory chain (refer to the information below), the administration of riboflavin and Coenzyme Q10 (CoQ) was started in addition to a 2‐line antiepileptic drug (AED) regimen during the third week after birth. Thiamine supplementation was initiated at 5 months of age after receiving a second opinion at another hospital. The patient exhibited microcephaly but did not experience any further deterioration. The nasogastric tube was removed on the thirteenth day, and the patient is currently receiving nutrition orally. Fidgety movements were not observed during follow‐up. By 6 months of age, the patient demonstrates increased alertness during daytime hours. Although he responds to auditory stimuli and exhibits spontaneous smiles, he does not exhibit a social smile, lacks eye contact, and does not reach to grasp objects. The patient has achieved head control, is able to roll from a prone to supine position but cannot sit without support. Antigravity strength is normal but spontaneous movement repertoire is poor and there is persistent hyperexcitability. The presence of four limbs pyramidal signs remained unchanged.

### Genetic analyses

**Figure 3:**
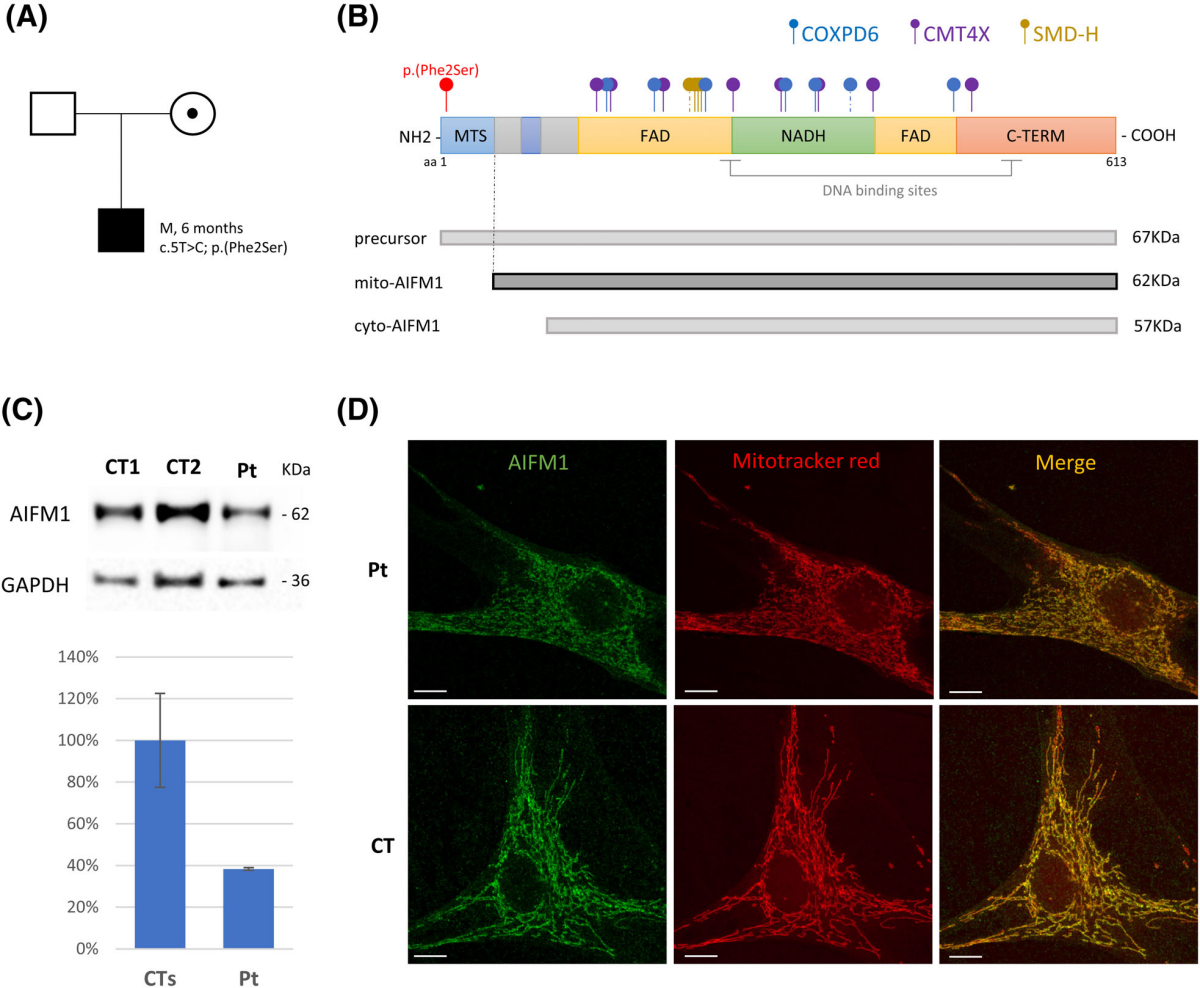
(A) Family pedigree and segregation analysis for the *AIFM1* c.5T>C variant. (B) Scheme of AIFM1 protein with its main domains and AIFM1 isoforms (precursor AIFM1, mitochondrial and cytosolic isoform) with their predicted molecular weights. Dot‐end arrows indicate the position of the novel p.Phe2Ser variant (in red) and of published *AIFM1* variants (with colors according to the corresponding phenotype). Dashed line were used for intronic variant affecting splicing junctions. (C) Immunoblot analysis of fibroblasts from patient (Pt) and controls (CT1, CT2) using antibodies against AIFM1 and GAPDH, the latter used as loading control. The graph reports percentages of the values of AIFM1/GAPDH signals obtained by densitometric analysis. One hundred per cent corresponds to the mean value from controls. (D) Representative images of immunofluorescence staining obtained with the anti‐AIFM1 antibody (green) and the mitochondrial marker Mitotracker (red) in fibroblasts from the patient (Pt) and a control (CT). The merged signals are reported in the right‐hand panels. Scale bar: 10 μm.

A rapid CES analysis revealed the presence of the rare variant (NM_004208.4) c.5T>C in the *AIFM1* gene, causing the missense substitution p.(Phe2Ser), reported only once in the reference population GnomAD (rs1326038976; 1/151242 alleles, in a female individual); therefore, we assigned the PM2 ACMG evidence. Pathogenicity prediction tools gave contrasting outputs (e.g., Polyphen: benign; SIFT: deleterious). The variant is predicted to affect the MTS region of AIFM1, hence possibly impairing its ability to localize in the inner membrane of mitochondria. Hence, we assigned the PM1 ACMG evidence. Moreover, because missense variants are a common mechanism of disease in *AIFM1* deficiency, with no truncating variants so far described, we assigned the PP2 ACMG evidence. At that point, according to the ACMG criteria, the variant was classified as VUS (Variant of Uncertain Significance).^19^ The variant was inherited from the healthy mother, who is heterozygous (Fig. 3A), in accordance with the *AIFM1* X‐linked recessive mode of inheritance. We also performed CGH array analysis to exclude genomic rearrangements in other regions, but no pathogenic variants were identified.

### Functional assays on patient fibroblasts

Respiratory chain analysis on cultured fibroblasts from the patient demonstrated a reduction in the activity of RC Complexes I, III and IV (36%, 48%, and 59% residual activity, respectively), as previously reported for other subjects harboring *AIFM1* pathogenic variants. Immunoblot analysis showed strong decreased amount of AIFM1 protein in patient's fibroblasts when compared with controls (Fig. 3C), indicating instability of the mutant protein. No immunoreactive material corresponding to the precursor protein was detected. Fluorescence studies corroborated these findings, with overall reduction of the AIFM1 mitochondrial‐localized signal and absence of cytosolic AIFM1 staining. These experimental data confirmed a deleterious outcome of the identified variant, causing reduced amount but not mislocalization of the mutant protein. Taking into account these well‐established functional results (PS3 ACMG evidence), we re‐classified the variant as Likely Pathogenic.^19^

## Discussion

AIFM1 is a flavoprotein with at least six functional domains, including an N‐terminal MTS, a hydrophobic transmembrane sequence, three structural domains that coordinate the binding of FAD and NADH molecules, and a C‐terminal motif (Fig. 3B). The soluble form of AIFM has been well‐known for its involvement in caspase‐independent cell death.^1^, ^24^ However, there is increasing evidence suggesting a stronger association between human pathophysiology and impaired mitochondrial bioenergetics rather than altered apoptotic pathways. Indeed, AIFM1 provides a significant contribution to the biogenesis and maintenance of RC complexes, primarily by favouring the import and oxidative folding of RC subunits in the intermembrane space.^4^ Whether AIFM1 is directly implicated in electron transfer to RC complexes, and what is the meaning of its interaction with other substrates or its role in other processes (such as mitochondrial Ca^++^ uptake) is less understood.^2^, ^25^ Of note, we cannot exclude that a variable combination of OXPHOS failure and increased susceptibility to caspase‐independent apoptosis (independent of RC dysfunction) may underlie the wide spectrum of presentations of *AIFM1*‐related disorders (Table S1).^5^

In our patient, hemizygote for the p.Phe2Ser likely pathogenic variant, we observed a combined oxidative phosphorylation defect affecting RC complexes I, III and IV. COXPD are usually caused by mutants in nuclear genes implicated in diverse mitochondrial functions including mtDNA maintenance, import of proteins into the mitochondrion, mitochondrial membrane biogenesis and mitochondrial transcription or translation.^26^, ^27^ AIFM1 has a known role in correct assembly and maintenance of the OXPHOS complexes. Accordingly, biochemical defects of different complexes (mainly I and IV) are common in *AIFM1* deficiency,^28^, ^29^ particularly in patients with the encephalomyopathic COXPD6 disorder but also in some CMT4X cases.

Early‐onset, drug‐resistant seizures have already been described in neonates harbouring *AIFM1* mutants (Table S2).^30^, ^31^ However, this is the first case presenting with diffuse white matter signal alteration and relative sparing of basal ganglia. We may speculate that partum‐induced stress exacerbated the bioenergetic failure that eventually led to such a diffuse involvement, which has not been reported in other cases. Of note, the clinical picture initially exhibited similarities to hypoxic–ischemic encephalopathy (HIE). However, the evolution in the clinical phenotype, the persistence of suppression‐burst pattern on EEG and the observed alterations in brain MRI and MRS were contradictory to a diagnosis of HIE. Hence, when there are discrepancies between clinical features and neuroradiological/neurophysiological findings, HIE mimics should be considered.^32^ In such cases it may be prudent to contemplate screening for COXPD along with other conditions such as molybdenum cofactor deficiency or sulfite oxidase deficiency.

In contrast to what is documented in other COXPD6 cases presenting within the 1st year of life, rapid progression and signs of muscle or peripheral nerve involvement were not observed during the six‐month follow‐up.^5^, ^16^ However, electromyography (EMG) was not performed to exclude the presence of axonal neuropathy and it is possible that features such as lower motor neuron degeneration, respiratory insufficiency, progressive dysphagia, ptosis, external ophthalmoplegia, and ataxia may manifest later.^33^ Additionally, due to the limited duration of the follow‐up, it is challenging to determine whether the observed lack of symptoms is a direct result of vitamins supplementation.

As previously mentioned, reduced RC activities are not exclusive of COXPD6, but can be also observed in some CMT4X patients, suggesting common pathogenic mechanisms. In 1985 Cowchock and colleagues first reported seven family members presenting from birth to late childhood with a slowly progressive, axonal neuropathy and a variable combination of hearing loss and cognitive impairment (CMT4X).^34^ Later reports expanded the phenotype including visual loss, color blindness,^9^ and more recently pure motor axonal neuropathy resembling hereditary motor neuropathies.^35^, ^36^ Intra‐familiar variability can be striking, adding to the complexity of the pathophysiological mechanisms underlying *AIFM*‐1 related disorders.

A predominant cerebellar phenotype characterized by limb ataxia, dysarthria, and abnormal eye movements (e.g., slow saccades) has been recently documented in patients encompassed within the spectrum of CMT4X (i.e., in presence of axonal neuropathy and hearing loss).^9^, ^10^, ^33^, ^37^ Cerebellar atrophy, mostly affecting the vermis, may develop with time and not be evident on first assessment. Sustained myoclonus was reported in one patient as well as external ophthalmoplegia.^10^ Of note, the latter feature was also present in a child presenting with a disease course characterized by alternating periods of stability and rapid progression who eventually developed respiratory failure, hence corroborating a disease continuum between COXPD6 and CMT4X.^33^

A peculiar combination of central hypomyelination and spondyloepimetaphyseal dysplasia (H‐SMD) constitutes a specific phenotype that only partially overlaps with other *AIFM1* cases.^13^ After a normal neonatal period, patients present within the first 2 years of life with developmental delay/arrest and predominant cerebellar signs (e.g., nystagmus and truncal ataxia). Cognitive impairment is variable, but significant disability mainly occurs because of progressive cerebellar, pyramidal and lower motor neuron degeneration (e.g., severe dysarthria, spasticity, muscle weakness and respiratory failure). Progressive skeletal deformities such as scoliosis likely contribute to the severe respiratory phenotype observed in H‐SMD. Visual loss associated with macular degeneration, achromatopsia, and optic nerve atrophy can be present, but no seizures were reported in these patients. Early death may occur within the second decade of life.

Genotype–phenotype correlations in *AIFM1*‐associated disorders are poorly defined; indeed, variants described in a specific clinical presentation may affect different domains, and variants in the same protein region (e.g., the FAD‐binding domain) may cause different phenotypes (COXPD6 or H‐SMD) (Fig. 3B, Table S1). However, there are some possible exceptions such as the variants reported in H‐SMD, which are found within the same narrow region of the gene (Intron 6 and exon 7) located at the end of the first FAD domain.^12^, ^38^

The reasons underlying phenotypic variability of *AIFM1*‐related disorders, even within the most severe end of the spectrum,^33^ remain elusive. Intriguingly, Diodato at al suggested that different amino acid substitutions within the same region may in fact exert a variable effect on the oxidized vs reduced state of the AIFM1 protein,^39^, ^40^ hence affecting not only its redox properties but also protein stability and conformational changes (i.e., monomeric vs dimeric). In addition to altered properties, various missense amino acid changes have been proved to cause protein instability in a subset of AIFM1 cases; the degree of AIFM1 reduction is therefore another possible modulator of phenotype spectrum and severity.^30^

The novel c.5T>C; p.(Phe2Ser) variant described here is the first variant in *AIFM1* located in the MTS. It putatively affects the ability of AIFM1 to correctly localize to the mitochondria; however, experimental studies did not show any evidence of impaired processing or mislocalization of the AIFM1 protein. Nevertheless, the steady‐state level of the mutant protein was clearly reduced compared to controls suggesting that the variant has a negative impact on mitochondrial import with subsequent degradation of the cytosolic precursor protein. Notably, the mature AIFM1 (without the MTS harbouring the variant) present inside mitochondria of our patient is expected to be identical to the mature protein in normal subjects; hence, its reduced amount (rather than any altered property of the mutant protein) can be considered responsible for the phenotype. Additional patients with *AIFM1* variants located in different domains (including other cases with variants in the MTS) are required to better define genotype–phenotype correlations in this X‐linked condition with such a spectrum of clinical pictures.

## Conclusions

AIFM1‐associated disorders encompass a wide array of clinical presentations involving degeneration of various components of the central and peripheral nervous systems. The unifying characteristic is the presence of axonal neuropathy, while the extent of involvement of the cerebellum, pyramidal tracts, extrapyramidal system, and lower motor neurons varies across the spectrum. Notably, respiratory dysfunction appears to be predominant, although with variable onset, in both COXPD6 and H‐SMD.

Seizures have only been reported in patients with COXPD6. This case report suggests that an AIFM1 defect should be considered in newborns exhibiting intractable neonatal seizures and radiological features resembling hypoxic–ischemic encephalopathy. To support the diagnosis, a punch skin biopsy should be considered, as a reduction in respiratory complex activity was demonstrated in cultured fibroblasts from all neonatal cases.

The reasons underlying such variability of phenotypes remain elusive and genotype–phenotype correlations are difficult to perform in the absence of functional studies. Gaining a better understanding of the disease mechanisms would facilitate the targeted use of supplements such as riboflavin or CoQ, whose efficacy in affected patients remains variable and unclear.

## Author Contributions

All authors fulfill the criteria of authorship and no one else who fulfills the criteria has been excluded. Alberto A. Zambon, Daniele Ghezzi, Cristina Baldoli, Gianni Cutillo: Drafting/revising the manuscript for content, including medical writing for content; study concept or design; acquisition of data; interpretation of data; Katia Fontana, Valentina Sofia: Drafting/revising the manuscript for content, including medical writing for content, acquisition of data, interpratation of data. Alessia Nasca, Stefano Vinci, Ivana Spiga, Eleonora Lamantea, Giovanna F. Fanelli: acquisition of data, interpretation of data. Paola Carrera: Drafting/revising the manuscript for content, including medical writing for content, interpretation of data; study supervision and coordination. Rosanna Rovelli, Antonella Poloniato, Maria Grazia Patricelli, Maria Grazia Natali Sora, Massimo Filippi, Graziano Barera: interpretation of data; study supervision and coordination.

## Conflict of Interest

The authors have no disclosures to report regarding the present study.

## Funding Information

The authors received no funding for the study.

## Supporting information


**Table S1.** Summary of clinical characteristics of AIFM1‐associated disorders.

**Table S2.** Summary of all the included studies reported specifically on epileptological features of the affected patients.
