# Supplementary material for: Expanding the spectrum of neonatal‐onset AIFM1‐associated disorders
Source: Ann Clin Transl Neurol. 2023 Aug 29;10(10):1844–53. doi: 10.1002/acn3.51876 (PMC10578896; doi:10.1002/acn3.51876)
Supplement: Supplementary file 1 — Table S1. Summary of clinical characteristics of AIFM1‐associated disorders. [file ACN3-10-1844-s002.docx]

**Table 1**. Summary of clinical characteristics of AIFM1-associated disorders.

|  | **Case (Patient, Age at last observation)** | **Genetic variant** | **Domain** | **Age of onset** | **Survival** | Hypotonia | Muscle weakness +/- atrophy | Developmental delay | Intellectual disability | Cerebellar Ataxia +/- other signs | Seizures | Microcephaly | Hearing loss | Pyramidal signs | Spondylometaphyseal dysplasia | Cardiomyopathy | Respiratory impairment | Peripheral neuropathy | Neurogenic changes on EMG | Elevated CK and transaminases | Lactic acidosis/Elevated lactates | **Brain MRI** | **Muscle Biopsy** | **OXPHOS Assay** |
| --- | --- | --- | --- | --- | --- | --- | --- | --- | --- | --- | --- | --- | --- | --- | --- | --- | --- | --- | --- | --- | --- | --- | --- | --- |
| Combined oxidative phosphorylation deficiency | **Present Case (P1, 6mo)** | c.5T>C; p.(Phe2Ser) | MLC | neonatal | alive | - | - | + | + | n/a | ++ | + | n/a | + | - | - | - | n/a | n/a | +/- | +/- | Diffuse WM involvement; High lactate peak | ND | ↓ activity of complex I,III and IV (fibroblasts) |
|  | Ghezzi et al 2010 (P1, 5y; P2 1 y) ^5^ | c.601_603; p.(R201del) | FAD-binding site | 1st year | P2 deceased at 16 mo | + | ++ | + | + | n/a | ++ | n/a | n/a | - | - | - | ++ | + | n/a* | n/a | + | Abnormal signal basal ganglia (at 1 y) | Dystrophic changes, no type grouping; mtDNA depletion.  ragged-red fibres, COX-SDH abnormalities. | ↓ activity of complex I, III and IV (fibroblasts) |
|  | Diodato et al 2016 (P1 3mo; P2, 6mo)^16^ | c.1013G>A; p.(Gly338Glu) | Regulatory 510–550 peptide region | neonatal | alive | + | ++ | + | n/a | - | + | n/a | n/a | - | - | - | ++ | + | ++ | n/a | + | Cortical atrophy + ventriculomegaly; High lactate at MRS | Neurogenic changes. COX deficiency | ↓ activity of complex IV (muscle and fibroblasts) |
|  | Peng et al 2022 (P1, 4mo; Brother with analogous presentation, 4mo) ^30^ | c.1164 + 5G>A | Intronic variant causing reduced AIF1 expression | neonatal | P1 deceased at 4mo, brother deceased at 4 mo | + | ++ | + | + | n/a | - | n/a | - | - | - | - | + | n/a | n/a | n/a | + | Abnormal signal basal ganglia + ventriculomegaly; High lactate at MRS | EM: abnormal mitochondria | ND |
|  | Berger et al 2011 (P1, 4mo; P2 , 4mo) ^15^ | c.923G-NA, p.(Gly308Glu) | NADH-binding site (amino acids 263–400) | intrauterine | P1 deceased at 4y PT 2 deceased at, 3 mo | ++ | ++ | ++ | n/a | n/a | ++ | n/a | n/a | - | - | ++ | + | n/a | n/a | - | - | Abnormal signal basal ganglia + choroid plexus cysts+ ventriculomegaly | Dystrophic changes. Severe COX deficiency | ↓ activity of complex I and IV defect (muscle) |
|  | Morton et al 2017 (P1, 4 mo) ^31^ | c.1436A>G; p.(Gln479Arg) | Binding pocket of FAD and NAD | intrauterine | P1 deceased at 4mo | + | + | + | n/a | n/a | ++ | n/a | n/a | - | - | - | ++ | + | + | n/a | ++ | Abnormal signal basal ganglia + later involvement of brainstem and occipital lobes | Mild morphological changes. EM: abnormal mitochondria | ↓ activity of complex I and IV, normal II and III (muscle) |
|  | Moss et al 2021 (P1, 8 days; P2, 2 days; P3, 39 days) ^17^ | c.506C>T; p.(Pro169Leu) | FAD-binding site | intrauterine | P1, P2 and P3 deceased respectively at 8, 2 and 39 days. | + | + | n/a | n/a | n/a | + | + | n/a | n/a | + | + | + | n/a | n/a | n/a | ++ | ND (Ventriculomegaly at ultrasound) | No morphological changes | ↓ activity of complex IV (muscle) |
|  | Kettwig et al 2015 (P1, 11 y) ^33^ | c.727G>T; p.(Val243Leu) | FAD-binding site | 2-3 years | alive | + | + | + | n/a | ++ | + | - | + | - | - | - | ++ | + | + | - | - | Mild cerebellar atrophy + unspecific T2 abnormalities in occipital lobes | No morphological changes. No ragged red fibers. Mild COX deficiency | ↓ activity of complex I and IV (muscle) |
| CMT4X spectrum | Cowchok 1985 and Rinaldi et al 2012 (7 pts from 1 family, 0-22y) ^34,6^ | c.1478A>T; p.(Glu493Val) | C terminal (reduced redox activity but no structural changes) | 0-14 years | alive (except 1) | + | ++ | + | +/- | - | - | - | + | n/a | - | - | - | + | + | - | - | Multiple punctate T2 hyperintensities in the supratentorial WM | Neurogenic changes. No ragged-red or COX/SDH abnormalities. EM: abnormal mitochondria | normal (muscle) |
|  | Pandolfo et al 2020 (P1, 13y; P2, 42y; P3, 42y) ^35^ | c.1195G>A, p.(Gly399Ser) | NADH-binding site | 2 years | alive | - | + | - | + | ++ | - | - | + | - | - | - | - | + | n/a | - | + | Progressive cerebellar atrophy | ND | ND |
|  | Bogdanova-Mihaylova et al 2019 (7 p from 1 family, 28-49y) ^9^ | c.1019T>C; p.(Met304Thr) | NADH-binding site | 1.5-34 years | alive | - | + | +/- | - | ++ | - | - | ++ | + | - | - | - | + | n/a | n/a | n/a | Subtle WM changes and cerebellar atrophy in 1pt. Mild cerebellar atrophy in 3. | ND | ND |
|  | Heimer et al 2018 (P1, 17y; P2, 11y) ^10^ | c.1019 T>C; p.(Met340Th); c.422C>T; p.(Thr141Ile) | FAD- binding site; DNA binding motif | 4-5 years | alive | + | - | + | - | + | - | - | + | - | - | + | - | + | n/a | - | - | Progressive cerebellar (mostly vermian) atrophy | ND | ND |
|  | Ardissone et al 2015 (P1, 39y) ^7^ | c.784G.A; p.(Gly262Ser) | DNA-binding site | 1-2 years | alive | - | + | + | + | + | - | - | + | - | - | - | - | + | n/a | - | + | Normal in childhood. Late-onset cerebellar atrophy (19 years) + mild cortical and thalamic atrophy (30 years) | Neurogenic changes. Ragged-red fibres | ↓ activity of complex I and IV (muscle) |
|  | Wang et al 2021 (5 pt from 1 family, 14-64y) ^8^ | c.513G>A; p.(Met171Ile) | Near regulatory 509-559 peptide region (Reduced activity) | 5-11 years | alive | - | ++ | - | - | - | - | - | - | - | - | - | - | + | + | - | +/- | Normal | Ragged-red fibers | ND |
|  | Hu et al 2018 (P1, 55y; P2, 56y) ^36^ | c.630C>G; p.(Phe210Leu) | First FAD-binding site | 14 years | alive | - | + | - | - | - | - | - | - | - | - | - | - | + | + | - | n/a | ND | ND | ↓ activity of complex I and III (fibroblasts) |
|  | Sancho et al 2017 (P1, 12y; P2, 8y) ^35^ | c.629T>C; p.(F210S) | FAD-binding site | 1-2 years | alive | - | + | + | - | - | - | - | - | - | - | - | - | + | n/a | n/a | n/a | ND | ND | ND |
| H-SMD | Mierzewska et al 2017 (7 pts from 2 families, 7-17y) ^12^ | c.710A>G; p.(Asp237Gly) | First FAD-binding site | 1-2 years | All patients deceased between 7-17 years | n/a | ++ | + | ++ | + | - | n/a | + | + | ++ | n/a | + | + | - | - | - | Diffuse hypomyelination; mild cerebral and cerebellar atrophy; Decreased choline and NAA levels; normal lactate | Non-specific changes (1pt) | normal (muscle) |
|  | Miyake N et al 2017 (12 pts from 6 families,3-37y) ^13^ | c.710A>G; p.(Asp237Gly); c.705G>C; p.(Gln235His); c.720C>T; p.(Asp240Asp); c.710A>T; p.(Asp237Val); c.697-44T>G | First FAD-binding site | 1-3 years | 5/12 alive. 7/12 deceased between 3-37 years | n/a | + | + | +/- | + | - | +/- | /- |  | + | - | + | +/- | n/a | n/a | n/a | Diffuse hypomyelination; cerebellum, basal ganglia and spinal cord relatively spared | ND | ND |
|  | Edgerley et al 2021 (P1, 10y; P2 17y) ^38^ | c.697-27T>G p. in intron 6; c.720C>T; p.(Asp240Asp) | First FAD-binding site | 0-18 months | alive with respiratory support | + |  | + | +/- | + | - | - | + | + | + | - | + | + | - | - | - | Diffuse hypomyelination; brain stem and cerebellum relatively spared (non-progressive) | Normal | ND |

*Abbreviations: EM, electron microscopy; MRS, Magnetic resonance spectroscopy; NAA,* *N-Acetylaspartate; n/a, not available; ND, not done; WM, white matter*
