# Supplementary material for: Expanding the spectrum of neonatal‐onset AIFM1‐associated disorders
Source: Ann Clin Transl Neurol. 2023 Aug 29;10(10):1844–53. doi: 10.1002/acn3.51876 (PMC10578896; doi:10.1002/acn3.51876)
Supplement: Supplementary file 2 — Table S2. Summary of all the included studies reported specifically on epileptological features of the affected patients. [file ACN3-10-1844-s001.docx]

**Supplementary material - Epileptological features**

Among the different phenotypes reported in patients with AIFM1 mutations, seizures and epilepsy have been reported exclusively in patients with oxidative phosphorylation deficits.

Data on seizures or epilepsy and EEG patterns, despite being relatively prominent features in the diseases, are only briefly mentioned by the published literature. *Supplementary Table 1* summarizes electroclinical data in the included studies. In our patient neonatal seizures were among the presenting symptoms and the EEG showed a disorganized background with multifocal electroencephalographic and electroclinical seizures both during wakefulness and sleep, later evolving into a burst-suppression pattern. Seizures were resistant to phenobarbital, phenytoin and levetiracetam so midazolam infusion was started. A partial reduction in seizure frequency was observed after the administration of pyridoxine, although there might be an underlying role of concomitant polytherapy. Neonatal seizures were reported in other four previous cases and in two of them phenobarbital was sufficient to achieve a good control over the seizures. Three patients reported an epilepsy onset after the first year of life, one of them after the third year, and, in two of these patients, seizures were only partially controlled with phenobarbital. The EEG was described for three patients showing a pattern of multifocal epileptic discharges and seizures. No data on sleep architecture were available. How epileptic manifestations and their severity may impact on the severity of the phenotype is yet to be determined.

| **Reference** | **Patient** | **Seizure onset** | **Seizures types** | **Treatment** | **Control** | **EEG** |
| --- | --- | --- | --- | --- | --- | --- |
| Ghezzi et al. 2010 | Pt1 | 15 months | Generalized | Phenobarbital | Partial control | NA |
| Diodato et al. 2015 | Pt.2 | NA | NA | NA | NA | Multifocal epileptic discharges in central, parietal, and occipital regions |
| Berger et al. 2011 | PtA | Neonatal | NA | Phenobarbital | Good control | Scattered fronto-temporal and parietal spikes |
|  | PtB | Neonatal | NA | Phenobarbital | Good control | NA |
| Morton et al. 2017 | Pt1 | 2.5 months | Focal | Phenobarbital, Gabapentin (not tolerated) | Incomplete control | 4 days: modest signs of encephalopathy |
|  |  |  |  |  |  | 2.5 months: diffuse encephalopathy, frequent left occipital sharp waves, right occipital sharp waves, no electrographic seizures |
|  |  |  |  |  |  | 3 months: seizures arising from the right occipital and left parieto-occipital regions, |
| Moss et al. 2021 | Pt3 | Neonatal | NA | NA | NA | NA |
| Kettwig et al. 2015 | Pt1 | > 3 years (the onset was not specified) | Generalized | NA | NA | NA |
| Ma et al. 2019 | 1 | 1 day | Focal | NA | Good control | NA |

***Supplementary table 1:*** *Summary of all the included studies reported specifically on epileptological features of the affected patients.*
